# Building a Comprehensive System of Services to Support Adults Living with Long-Term Mechanical Ventilation

**DOI:** 10.1155/2016/3185389

**Published:** 2016-04-18

**Authors:** David Leasa, Stephen Elson

**Affiliations:** ^1^Respirology/Critical Care, London Health Sciences Centre and Western University, London, ON, Canada N6A 5A5; ^2^London Health Sciences Centre and St. Joseph's Health Care London, London, ON, Canada N6A 5A5

## Abstract

*Background.* Increasing numbers of individuals require long-term mechanical ventilation (LTMV) in the community. In the South West Local Health Integration Network (LHIN) in Ontario, multiple organizations have come together to design, build, and operate a system to serve adults living with LTMV.* Objective.* The goal was to develop an integrated approach to meet the health and supportive care needs of adults living with LTMV.* Methods.* The project was undertaken in three phases: System Design, Implementation Planning, and Implementation.* Results.* There are both qualitative and quantitative evidences that a multiorganizational system of care is now operational and functioning in a way that previously did not exist. An Oversight Committee and an Operations Management Committee currently support the system of services. A Memorandum of Understanding has been signed by the participating organizations. There is case-based evidence that hospital admissions are being avoided, transitions in care are being thoughtfully planned and executed collaboratively among service providers, and new roles and responsibilities are being accepted within the overall system of care.* Conclusion.* Addressing the complex and variable needs of adults living with LTMV requires a systems response involving the full continuum of care.

## 1. Introduction

Long-term mechanical ventilation (LTMV) is used for life extension and/or quality of life. The interface for application may be invasive (tracheostomy) or noninvasive (mask or mouthpiece); furthermore, it may be intermittent (nocturnal) or continuous in use to meet one's care goals. In Canada, persons using this technology may be residing in an intensive care setting or weaning centre [1], in another type of institutional setting [2], or in a community setting [3] (e.g., at home or specialized assisted living). Venues of care may be limited outside of an acute care hospital, if home is not an option [2]. When home is an option, multiple barriers exist that prevent safe and timely transitions and ongoing care, including lack of limited paid caregivers and community services, lack of transition programs, and difficulties in negotiating public funding [3]. Care coordination can be challenging given complex respiratory care technology, heterogeneous chronic diseases (e.g., degenerative neuromuscular diseases, thoracic restriction, central respiratory control disturbances, obesity-hypoventilation syndrome, and spinal cord injury), and coexisting functional and cognitive disabilities. Given this level of complexity coordinating care across multiple providers, facilities, and support services can become an enormous challenge for a healthcare system. More concerning is that the respiratory and other care needs of patients with LTMV may be neither identified in a timely manner nor adequately met.

In the South West Local Health Integration Network (LHIN) in Ontario we have partnered with multiple organizations to develop a system of care for adults requiring LTMV and those “at risk” of becoming so. Our vision has been an integrated and responsive system of interprofessional care, with defined roles and processes that extend outside the walls of our intensive care units and hospitals, to provide timely identification and safely transition of technology-dependent patients back to their communities and homes (if possible) but also to ensure continuing access to quality ventilator-associated care. We have envisioned an integrated system of patient care “that is coordinated across professionals, facilities, and support systems; continuous over time and between visits; tailored to the patients' needs and preferences; and based on shared responsibility between patient and caregivers for optimizing health” [4].

In the fall of 2012, seven organizations that shared a common interest in improving care for adults living with LTMV met to talk about how they could work better, together. This involved the London Health Sciences Centre (LHSC), a tertiary acute care and teaching hospital; St. Joseph's Health Care London (St. Joseph's), a tertiary ambulatory care, mental health, rehabilitation, and complex continuing care hospital; Grey Bruce Health Services, a community hospital with six hospital sites and a Level 3 ICU in its Owen Sound (Ontario) location; the South West Community Care Access Centre (CCAC), an agency mandated to provide community-based health services, Long-Term Care Home placement coordination and information and referral services; Participation House Support Services (PHSS)-London and Area, a nonprofit, community-based organization that provides assistance to people with significant physical and/or developmental disabilities through residential, day program, respite, and social and recreational services; Glendale Crossings, a Long-Term Care Home; and the South West Local Health Integration Network (LHIN), a provincial agency created by the Ontario Ministry of Health and Long-Term Care (MOHLTC) to plan, integrate, and fund local health care. Each of these organizations has a different but complementary role to play in serving adults with LTMV. As a result the group defined the population they had in common, created a shared vision, identified desired outcomes, and defined deliverables based on a three-phase process. The undertaking was considered a project to improve the quality of care coordination.

## 2. Methods

Our population of interest was adults living with respiratory failure regardless of cause, who required LTMV (invasive or noninvasive) and needed ongoing support and care to enjoy the best quality of life available. We envisioned a coordinated and integrated system of care that would

1. provide safe, high quality standards-based care,
2. support living in the most appropriate, least restrictive setting possible, but with the care and services needed,
3. support individuals and their families through life and living transitions as seamlessly as possible,
4. be able to adapt and change in light of changing needs,
5. adopt and teach healthcare professionals and caregivers best practices.

Our desired outcomes were to

1. identify the continuum of care needed by persons dependent on LTMV in terms of the living settings best suited to address and support their different and changing needs, the healthcare services and professionals required to provide the level of care needed in different settings, and the resources (especially human) needed to support the continuum of care as defined,
2. identify the structure and organization of services needed to manage and coordinate the delivery of care throughout the continuum including trouble shooting and crisis management, managing transitions, planning for and anticipating future system demands, and developing, introducing, and adopting changes to the standards of care throughout the continuum on an ongoing basis,
3. develop specific proposals and advocate for changes to support the system of care that is developed and endorsed by the participating organizations, their patients/clients/residents, and their families,
4. ensure that the system of services as defined and implemented functions effectively, with responsibility, and in the best interests of the people served,
5. define a framework to identify, collect, and present performance indicators that would best characterize our system of care and provide quantitative evidence of its impact.

The project was undertaken in three phases: (1) System Design, (2) Implementation Planning, and (3) Implementation. Phase 1 deliverables included a clearly defined and agreed upon vision, model of the continuum of care, and model of the operational processes needed to support system-wide quality of services, transitions, continuity, and coordination of care. Phase 2 deliverables included a resource needs analysis and plan to implement the continuum of care and operational model as defined. Phase 3 deliverables included implementation, ongoing operations, and sustainability, including the structure, processes, and resources to support ongoing evaluation, improvements, and planning for the future.

### 2.1. Phase One: System Design

**Figure 1:**
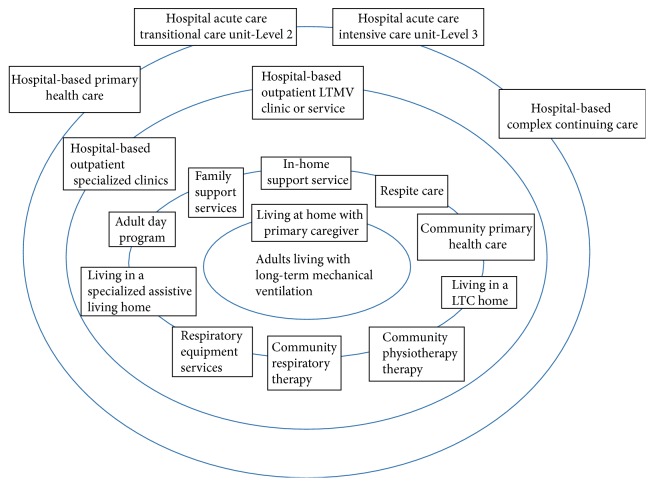
People, settings, and services for LTMV.

**Figure 2:**
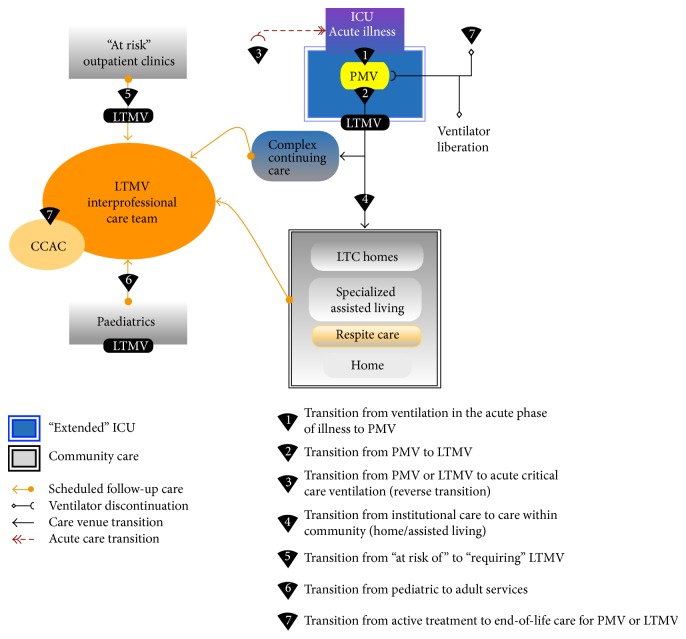
Systems model for care transitions and planned encounters.

This phase of the project took about a year to complete. The product was a comprehensive outline of the people, services, and settings needed to serve the LTMV population including respective roles and responsibilities in a fully coherent system of care, a description of the current state, and recommendations for the future [5]. It also addressed service coordination and planning, system leadership and accountability, and system performance assessment. This early work set the stage and framework for moving forward. It created a common focus and plan for action.  Figure 1 provides a graphic illustration of the components that we identified during this process. In concentric circles moving outwards, are those people, settings, and services most directly and consistently involved in the person's life and with their primary caregivers? The person living with LTMV is at the centre. Hospital-based services occupy the outer two rings. Outpatient services serve an ongoing role in the person's life in the community and are therefore closer to the person's life than hospital inpatient services. Inpatient services are designed to be time limited and to be able to move the person back into the community. The most highly specialized medical service is the hospital intensive care or transitional care unit. It has highly skilled medical and allied healthcare expertise to intervene when physiologic stability for ongoing community care is lost.  Figure 2 illustrates high-level patient flow maps to describe anticipated transitions in patient movement from one type or level of service to another. We envisioned the need to structure care coordination at various encounter points for the LTMV patient and used a recently defined lexicon [6] to help communicate and expedite patient care transitions. In addition to being able to describe and define the different settings and services that make up the system of health and supportive care services for adults with LTMV it is important to be able to describe how they connect and how people move through and interact with the system. Central to this map are the professionals with expertise to care for this population, regardless of where the person is residing at any point in time.

From a system of care perspective it is the transitions in care and the planned encounters that define how well the system is functioning “as a system.” Hallmarks of how well the system is working can be defined by the timing and appropriateness of the transitions (including how well they are planned and executed) and by the quality of relationships with clients and their families, and amongst the participating service providers and their organizations [7]. The nature of the conditions for adults living with LTMV necessitates an ongoing relationship and involvement with health and supportive care services, although the frequency and intensity of this involvement can be highly variable. Nevertheless, movement between services, involvement with multiple services at the same time, and transitions from one set or type of service or setting to another are a common pattern.

Two transitions warrant special attention. One of these is the transition from paediatric to adult services; the other is the transition to palliative and end-of-life care. In both transitions major life changes are taking place and there are significant vulnerabilities, and systems of care are challenged to respond well.

The transition from paediatric to adult services is a critical one in the lives of children/adolescents and their families. It is important to acknowledge the transition process itself. Parents and families develop an intimate relationship with their care providers as they jointly work to manage and address the complex health needs of their child/adolescent. Parents develop an intuitive understanding of their child's needs that are communicated and respected by care providers. Losing this relationship can be a significant loss filled with uncertainty and fear. There is a significant difference between the services offered to children and what is available to adults (in many ways they are different worlds) and unfortunately the transition can be experienced as a significant loss rather than as simply a transition. For example, as children a central contact for primary healthcare purposes is a paediatrician. As they become adults, paediatricians are no longer in a position to care for them, and yet, in most cases a family physician has not been involved. There is a constant challenge to address the primary care needs of these individuals, as adults. Standardized transition programs are needed to improve patient satisfaction [8].

People who need support and assistance to live with LTMV are often compromised in terms of their overall health status and life expectancy. Life expectancy can vary depending on the underlying condition and circumstances that have led them to need this kind of support. As with any person's life, individual circumstances are unique and outcomes are difficult to predict. At the same time, there is a responsibility and accountability on the part of the healthcare system to provide vulnerable persons, especially those who live with a dependency on LTMV and their family, timely access to palliative care professionals, including the alleviation of symptom distress, communication about goals of care, and support for end-of-life care.

Many of our LTMV patients have amyotrophic lateral sclerosis (ALS). Noninvasive ventilation (NIV) can palliate symptoms of respiratory failure, improve quality of life, and modestly increase survival. Our system was designed to offer and provide NIV and cough augmentation devices early in the care process using defined criteria. However, there is little information to inform how this technology in advanced ALS impacts end-of-life decision-making and the trajectory of dying [9]. Although invasive ventilation remains the only means to prolong the life of individuals with ALS by many years the final decision to continue NIV or to convert to tracheostomy must respect the person's autonomy. Open communication and clear identification of important issues relating to end-of-life decision-making (i.e., person's priorities and life plans) must occur. Optimum palliative management that incorporates hospital-based interprofessional care with community-based intervention is needed.

### 2.2. Phase Two: Implementation Planning

This phase took about nine months, to June 2014. It built on the concepts and recommendations developed in Phase One but involved a higher level of specificity and operational considerations. Participating organizations were expanded to include Grey Bruce Health Services (GBHS), a community hospital system with six hospital sites and a Level 3 ICU in its Owen Sound location.

Implementation Planning was organized around five main themes as follows:

1. Administrative oversight: we defined a System Oversight Committee, to provide system-level governance and leadership (with patients and families being invited members) and an Operations Management Committee, to address system-wide operational issues and needs.
2. Clinical standards: “best practice” clinical standards and guidelines [10, 11] were used as the standard of care for this population across the healthcare system. Process flow maps were used as key reference documents to plan, execute, and review transitions.
3. Business case development: basic services were advocated for, including access to community respiratory therapy services and nurse practitioners to support individuals living in the community across the South West LHIN. Complex continuing care was positioned as a specialized institutional venue, albeit having limited capacity, for those LTMV patients who were medically complex with physiologic stability but unable to return to community; acute care (Level 3 ICUs) was positioned to support LTMV patients in the community who needed temporary acute intervention when physiologic stability was lost and to assist in the transition to less intensive care when physiologic stability was reestablished.
4. Coordinated access: the South West CCAC mandate was to collaborate with others to facilitate access to the most appropriate community and post-acute hospital services needed by this population and to establish strong working relationships with primary care services to ensure continuing access.
5. Monitoring: the ongoing ability to monitor system improvements for this population was considered essential to the oversight structures. This included health system utilization information to help identify future service needs and to plan for changes in resources, especially community services. We utilized our partnership with the South West LHIN and their developing implementation of a Regional Integrated Decision Support solution (RIDS) to extract data that was routinely collected from participating providers including hospitals and the South West CCAC. RIDS is a data warehouse that collects and links data from a number of different data systems and allows analysts to track one individual's utilization across much of the healthcare system.

Implementation Planning produced a number of detailed flow maps that were created to serve as interorganizational protocols as to how different types of transitions should be addressed. These transitions included long-term and short stay scheduled transitions and short-term unscheduled transitions. It was decided that, rather than having a standing committee to manage person-specific transitions, responsibility and accountability for planning, executing, and reviewing transitions would involve those agencies and front-line staff directly involved with the individual and their family.

### 2.3. Phase Three: Implementation

This phase began in September 2014 and it activated the Oversight Committee, the Operations Management Committee, the collection and analysis of system performance measures, the coordination, planning, and implementation of specific transfers of individuals, and the implementation of new community programs and services thanks to funding made available by the South West LHIN. New programs included the introduction of funded community respiratory therapy services through the CCAC and the development of overnight respite care, specialized adult day programs, and new specialized residential supports by Participation House Support Services.

This phase also involved the development of a Memorandum of Understanding among the participating organizations that expanded to include the Huron Perth Healthcare Alliance (HPHA). HPHA is an alliance of four hospitals with a Level 3 ICU in its Stratford, Ontario location. This signed agreement secured a collective commitment to the provision of an integrated system of LTMV care across the LHIN.

## 3. Results

The system that is currently operational reflects the vision and objectives originally set out in 2012. Central to our system is a regional interprofessional LTMV outpatient clinic that provides access to specialized ventilator and respiratory care but also ensures care continuity.

Outcomes to date are as follows:

 enhanced CCAC service delivery:
   (i) community access to funded community RRT services,
   (ii) community access to nurse practitioners for invasive LTMV;
 regional interprofessional LTMV outpatient clinic for care continuity, including the following:
   (i) referral agreements with NMD and MND clinics, for timely care,
   (ii) NIV initiation in the clinic setting (prescription development and client training),
   (iii) scheduled encounters for ventilator prescription review,
   (iv) access to RRT, SLP, physiotherapy, nutritionist, palliative care, and CCAC,
   (v) RRT ventilator prescription modifications by telephone;
 creation and use of an equipment repository for NIV initiation and cough augmentation technology, pending VEP approval (previously a 4-week lag);
 clinician access to a regional (LHIN) health-information system (ClinicalConnect®) for information sharing, including CCAC clinical notes;
 ongoing development of a single, comprehensive, computerized information management system to track performance measures and drive continuous quality improvement (Regional Integrated Decision Support-connecting South West Ontario);
 memorandum of understanding signed amongst the participating organizations;
 increased LTMV community capacity through supportive housing and assisted living for complex/medically fragile, including respite (Participation House Support Services);
 early identification of the patient in the ICU for PMV and LTMV transitions.

New services have been funded by the LHIN that have been critical to moving the system forward. This has included building community capacity in the form of day programs, overnight respite care, specialized residential support services, and community respiratory therapy services. There are both qualitative (see Case Report) and quantitative (Table 1) evidences that a multiorganizational system of care is now operational and functioning in a way that previously did not exist.

**Figure 3:**
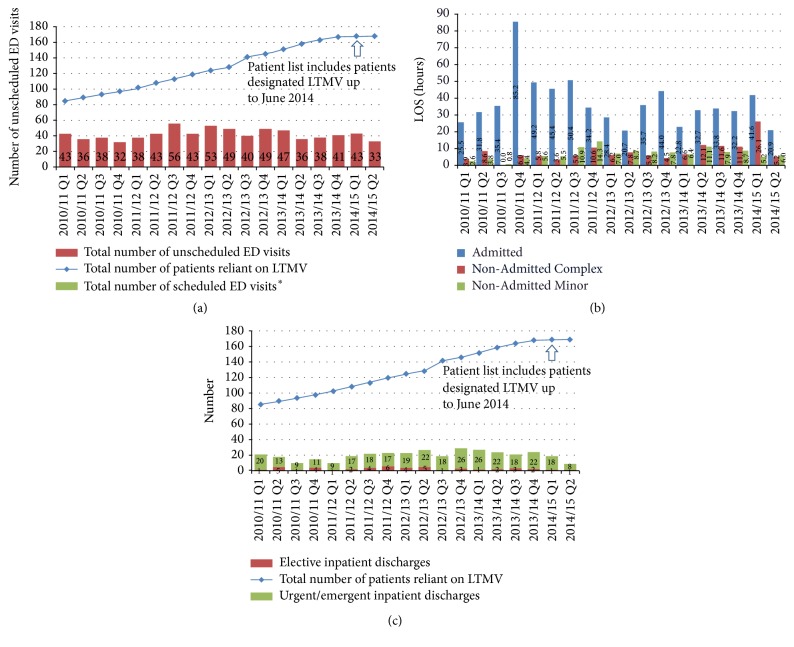
(a) Emergency department utilization (ED visits). Total number of ED visits for all patients reliant on LTMV, by ED visit type. ^*∗*^Note: from Q1 2010/11 until present, there have been no scheduled ED visits within this cohort. (b) Emergency department utilization (ED LOS). Length of stay (LOS) in ED (hours) for patients reliant on LTMV, 90th percentile. Note: the data above includes ED LOS in any facility that a LTMV patient visited. For comparison, in Q2 2014/15, Ontario, as a whole, had an Admitted LOS of 29.5 hours, a Non-Admitted Complex LOS of 6.8 hours, and a Non-Admitted Minor LOS of 4.0 hours. (c) Acute inpatient utilization (hospital discharges). Total number of acute inpatient discharges for all patients reliant on LTMV, by inpatient visit type.

System performance measures have been developed and some are being reported on a quarterly basis (Figures 3(a), 3(b), and 3(c)). There is case-based evidence that hospital admissions are being avoided, transitions in care are being thoughtfully planned and executed collaboratively among service providers, and new roles and responsibilities are being accepted within the overall system of care. It is anticipated that the system of services that has been created will continue to evolve and new needs will continue to be identified.

*Case Report.* In August 2007, a 23-year-old woman with congenital fibre-type disproportion myopathy was admitted to the ICU with hypercapnic respiratory failure and pneumonia. Preexisting problems included slowly progressive myopathy, mild cognitive delay, scoliosis, obesity (BMI 34), and moderate oropharyngeal dysphagia. Prior to admission she was living at home with her parents and was wheelchair dependent. Tracheostomy and percutaneous feeding tube insertion were performed to facilitate transition to nocturnal ventilation and then care at PI-CCC in December 2007. She required brief readmission to the ICU in January 2008 for pneumonia but returned to care at PI-CCC. In June 2008 she was discharged home following weight loss, feeding tube removal, decannulation, and transition to nocturnal oronasal mask NIV. Readmission to ICU occurred 2 years later in August 2010 again requiring intubation for hypercapnic respiratory failure with pneumonia. Significant weight gain had complicated her course. Repeat tracheostomy insertion was performed. She was transferred back to care at PI-CCC in December 2010 with the same invasive ventilator care as previously. In January 2012 she was transitioned to supportive housing/assistive living in the community at PHSS. She required a brief ICU admission to transition to a new ventilator (obtained from the VEP) and training was provided to her support workers to safely meet her nocturnal tracheostomy (uncuffed) ventilation and cough augmentation needs. Ongoing care includes scheduled visits to the regional interprofessional LTMV outpatient clinic for ventilator prescription review and monthly visits by a community RRT (for tracheostomy change) and a CCAC community nurse practitioner. Since 2012 she has required only one brief ICU admission for constipation and fecal impaction.

## 4. Discussion

Significant variability exists across jurisdictions in Canada in the transition and ongoing care of LTMV patients [12]. Although some have developed selective innovations to improve care, many fail to address all aspects of integration including functional (e.g., oversight, strategic planning), organizational (e.g., ownership, contractual agreements), professional (e.g., formal collaboration amongst clinicians), and clinical integration (e.g., delivering needed services) [4]. In Ontario, the Ministry of Health and Long-Term Care has emphasized priorities for LTMV [13]: avoid, wherever possible, hospital admissions due to respiratory failure for those “at risk” of long-term invasive ventilation; help those who have been admitted to hospital return to their community; and provide the supports and services needed for the individual to stay in the community safely, and for as long as possible. In the South West LHIN we have responded by building a comprehensive system of services to support adults living with LTMV using a three-tiered approach of System Design, Implementation Planning, and Implementation. Now functional, our Oversight Committee brings senior organizational leadership to the table, our Operational Management Committee brings operational leaders together to address operational issues, and case specific transitions groups focus on transitional needs as transitions are planned, prepared for, and implemented (Table 2). While all three groups have specific roles and responsibilities they are complementary to one another and reinforce their common commitment to excellence, partnerships, and care coordination.

Our work continues. Ongoing endeavours include continuing discussions with long-term care homes for caring of the older patient requiring noninvasive ventilation, increased collaboration with community palliative care services including end-of-life care for the patient on mechanical ventilation, and demonstrating that a comprehensive database can be used to inform decision-making and system planning for the LTMV population within the South West LHIN. The latter explores linkage to the Ontario Ventilator Equipment Pool [14] user database to identify all community-based users of mechanical ventilators in the South West LHIN and use this data to inform RIDS-based analysis. Further work will define those performance indicators, beyond the utilization of acute care services, that best measure the healthcare quality of our system (i.e., performance indicators that are standardized, valid, and reliable). These will include indicators that aim to measure care coordination (e.g., care within and across teams, care continuity, linkage to community services, and timeliness of care) and patient/client and caregiver satisfaction.

In a recent review five major themes emerged to define care coordination, where it involves numerous participants, is necessitated by interdependence among participants and activities, requires knowledge of others' roles and resources, relies on information exchange, and aims to facilitate appropriate healthcare delivery [15, 16]. As a system of services, no one person, service, agency, or organization can serve the health and supportive care needs of this population by working in isolation. Creating, sustaining, and growing relationships through partnerships are the lifeblood that will create, foster, and strengthen service delivery, system coordination, planning, and leadership. The organizations involved with this initiative have a long history of collaboration and partnership development based on a shared commitment to integrate health services across organizations so patients/clients can access the most appropriate level of care on a timely basis. For example, CCAC and all hospitals in the South West LHIN have a signed shared accountability agreement that defines their respective roles and responsibilities that has been operational for almost 10 years.

The development of a systems model of care at the local and regional level helped to define and refine roles and responsibilities with respect to serving the LTMV population. It allowed everyone involved acknowledging his or her role in the continuity of care. The model of care defined not only the component parts of the system but also how people interact and support others as they move into and participate in the system.

## Figures and Tables

**Table 1:** Selected program characteristics.

*South West LHIN*		
(i) Approximately 200 health service providers, including 20 hospital corporations across 33 sites		
(ii) Area from Lake Erie to the Bruce Peninsula and home to ~ one million people		
(iii) 8 hospital sites with Level 3 ICUs totaling 97 ventilator care beds (68% in London)		
(iv) Regional programs for Neuromuscular and Motor Neuron Diseases in London		

*Prolonged Mechanical Ventilation, MSICU, University Hospital-LHSC*		
PMV (≥21 days on a ventilator) represents 4.6% of MSICU admissions but 38% of total ICU patient days		

Alternate Level of Care (ALC) is defined as a ventilated patient occupying an ICU bed but not requiring the intensity of resources/services provided in this care setting. Patients declared ALC in the MSICU has decreased over the past 3 years		

Date	Number of ALC Days	Number of patients declared ALC

2012-13	588	6
2013-14	108	3
2014-15	187	2

*LTMV, Institutional Care*		
*Parkwood Institute Complex Continuing Care, as of April 2015*		
6 beds (100% occupancy; median LOS 1053 days with range of 136–1983 days).		

*Regional Interprofessional LTMV Outpatient Clinic 2010–2015*		

Primary reason for LTMV	Number of active clients (as of July 2015)	Number of inactive clients (2010–2015)

Noninvasive ventilation	139	133
Neuromuscular disease	97 (27 ALS)	199 (95 ALS)
Chest wall restriction	4	1
Complex OSA/OHS	34	13
Central apnea	4	0
Invasive ventilation	20	12
Community	14	
PI-CCC	6	

Outpatient NIV starts		
Year	#	

2010	3	
2011	18	
2012	29	
2013	30	
2014	29	
2015 (6 months)	17	

**Table 2:** Organizational structure characteristics.

	Oversight Committee	Operations Management Committee	Transitional care teams
Leadership	(i) Organization leaders (ii) Strategic (iii) Vice presidents or senior directors (iv) Chair and Vice-Chair model	(i) Organization leaders (ii) Operational (iii) Clinical directors, program managers (iv) Co-Chair model	(i) Clinical leaders, operational leaders, and clinical specialists with direct client/patient responsibilities (ii) Chair changes with case being addressed

Leadership attributes	(i) A systems thinker who is able to build consensus among participants and flag strategic issues (ii) Excellent meeting management skills	(i) An operational leader who is able to build consensus among participants and flag operational priorities (ii) Excellent meeting management skills	(i) A clinical/operational leader who is able to problem-solve and engage senior leaders in the solution as needed

Membership	(i) All member organizations	(i) All member organizations	(i) People within the “circle of care,” family members and client/patient

Physician leadership	(i) Respirologist	(i) Respirologist	(i) Physician involved on a case specific basis

Decision-making	(i) By consensus	(i) By consensus	(i) By consensus

Support roles	(i) Administrative support donated by Chair's agency (ii) Project management support	(i) Administrative support donated by Co-Chair's agency, shared (ii) Project management and decision support for performance reporting	(i) Supported by lead organization for a particular case

Terms of reference	(i) Yes; defines mandate, role & responsibilities	(i) Yes; defines mandate, role, and responsibilities, reporting to the Oversight Committee	(i) No; but process flow maps have been created to guide decision-making processes (ii) Escalates system issues to the Operations Management Committee

Level of organizational commitment	(i) High (ii) CEO has signed off on a Memorandum of Understanding; reinforcing continuity as individuals change	(i) High (ii) Participants involvement is supported and sanctioned by their organization	(i) High (ii) Participant involvement is supported and sanctioned by their leaders

## Abbreviations

RRT: Registered respiratory therapist
LTMV: Long-term mechanical ventilation
NMD: Neuromuscular disease
MND: Motor neuron disease
VEP: Ontario ventilator equipment pool
CCAC: Community Care Access Centre
SLP: Speech language pathologist
PMV: Prolonged mechanical ventilation
ICU: Intensive care unit
NIV: Noninvasive ventilation
PI-CCC: Parkwood Institute Continuing Complex Care
PHSS: Participation house support services
MSICU: Medical Surgical Intensive Care Unit, University Hospital
OSA/OHS complex: Obstructive sleep apnea/obesity-hypoventilation syndrome
CCC: Complex continuing care
LTC: Long-term care home.
